# Prevalence and associated factors of premenstrual dysphoric disorder among high school students in Finote Selam town, northwest Ethiopia

**DOI:** 10.3389/fpsyt.2024.1362118

**Published:** 2024-06-21

**Authors:** Getasew Kibralew, Demeke Demilew, Selam Koye, Sewbesew Yitayih, Mulualem Kelebie, Mamaru Melkam, Gebresilassie Tadesse, Setegn Fentahun, Girum Nakie, Yilkal Abebaw Wassie, Tadele Amare

**Affiliations:** ^1^ Department of Psychiatry, School of Medicine, College of Medicine and Health Science, University of Gondar, Gondar, Ethiopia; ^2^ Department of Medical Nursing School of Nursing, College of Medicine and Health, Science, University of Gondar, Gondar, Ethiopia

**Keywords:** premenstrual dysphoric disorder, high school students, prevalence, associated factors, Ethiopia

## Abstract

**Background:**

Premenstrual dysphoric disorder (PMDD) is the most prevalent but neglected psychiatric disorder, with somatic symptoms that are severe enough to markedly affect usual daily activities and have a negative impact on mental health and quality of life by affecting female patients’ behavior and cognition. Studies regarding premenstrual dysphoric disorder and associated factors among high school students in low- and middle-income countries are limited. Therefore, the aim of this study was to assess the prevalence and associated factors of PMDD among high school students, and this is pivotal in further investigation.

**Methods:**

A school-based cross-sectional study was conducted from March 25 to April 17, 2023 using a simple random-sampling technique to select a sample of 564 participants. Premenstrual dysphoric disorder was assessed using the Diagnostic and Statistical Manual of Mental Disorders (DSM-5). Self-administered standardized questionnaires were used to collect data.

**Result:**

A total of 548 study participants participated, with a 97.2% response rate. The prevalence of premenstrual dysphoric disorder among high school students was found to be 33.03% (95%CI: 29.20–37.09). In a multivariable analysis, irregular menstruation cycle (AOR = 4.242, 95%CI = 2.182–8.246), depression (AOR = 5.272, 95%CI = 2.779–10.002), having greater than 4 days of menstruation bleeding duration (AOR = 2.138, 95%CI = 1.105–4.138), and high perceived stress (AOR = 3.468, 95%CL = 1.217–9.880) were the factors significantly associated with premenstrual dysphoric disorder.

**Conclusion:**

The overall prevalence of PMDD which was one-third among high school students was high. Moreover, long duration of menstruation bleeding, depressive symptoms, irregular menstruation cycle, and high perceived stress were significant factors in PMDD. Therefore, it needs early screening and intervention in primary healthcare settings, especially for those who have high perceived stress, having depression, having a long duration of menstruation bleeding, and having an irregular menstruation cycle, so as to have good academic achievement and psychological wellbeing.

## Introduction

Premenstrual dysphoric disorder (PMDD) is a combination of physical pain and emotional or behavioral abnormalities that start around a week before the start of menstruation (1). Around the world, 3% to 8% of women of reproductive age experience premenstrual dysphoric disorder (PMDD) (2). Prevalence data from around the world reveals that up to 75% of all women of reproductive age may suffer premenstrual syndrome (PMS), with 5% to 20% of women of reproductive age reporting moderate to severe premenstrual complaints. About 94.8% of women of reproductive age had PMS, characterized by one or more physical, emotional, or behavioral symptoms in the days leading up to menstruation (15 to 49 years) (3). In Africa, including Ethiopia, the magnitude of premenstrual dysphoric disorder is between 10.2% and 66.9% (4–7).

Premenstrual dysphoric disorder (PMDD) is a serious condition that seriously impairs a woman’s functionality and quality of life. It affects female behavior, cognitive capacities, mental health status (like being seven times more likely to have suicidal thoughts and nearly four times more likely to attempt suicide), academic performance, interpersonal relationships, daily activities, and work productivity. A woman’s general physical health can all be negatively impacted by PMDD (8–11). Even though the majority of women with PMS can carry out their daily tasks when in its extreme form, this condition has been linked to increased absenteeism from work and school, poor academic performance, high suicide ideation and attempt rates, and significant mental health issues (5, 12).

Students struggle to get out of bed and be at class on time, and unexpected mood changes make it challenging to deal with the fallout. When PMS is not treated and women experience additional personal or environmental stress, the symptoms become more severe, eventually developing into PMDD, a mental disease (13–15).

Different factors affect PMDD, including age, body mass index (BMI <30 kg), monthly pain, amount of menstrual blood loss, history of physical and mental illness, treatment-seeking behavior, history of traumatic events, sleeping hours, physical activity, not using the family planning method, and maternal history of PMS (16–18). Blood loss during periods, the existence of dysmenorrhea, a mother or sister with a positive premenstrual syndrome, and low agreeableness and extroversion as well as high neuroticism were significant risk factors for PMDD in terms of personality traits (10, 19, 20).

A family history of PMS and dysmenorrhea increases the risk. Other menstrual characteristics, such as age at menarche, irregular menstruation, a longer average menstrual cycle, menorrhagia, consuming tea, coffee, sweets or sweetened beverages, and junk food, food intake, and stress were significant predictors of PMDD (4, 10, 21).

While PMDD among high school students has been relatively well researched in developed countries, more premenstrual syndrome studies are available in developing countries, including Ethiopia. Nevertheless, in Africa, including Ethiopia, some premenstrual dysphoric disorder studies are available among university students. According to my research engine, a few studies were conducted among high school students in developing countries. Even though premenstrual disorders (PMDD) have a significant impact on academic performance, yet, in Ethiopia, premenstrual difficulties still receive insufficient attention (19). Those few studies were done without including factors like perceived stress and clinical factors like a history of mental illness (suicide and major depression). Therefore, this study assessed the prevalence of PMDD and various factors that might lead to early interventions for further obstacles among high school students.

## Methods and materials

### Study area, design, and period

The study was conducted in west Gojjam. Finote Selam town is one of the zones in the Amhara Region of Ethiopia. It is far by 171.2 km from Bahir Dar of Amhara Region town and 246 km from Addis Ababa, the capital city of Ethiopia. Based on the 2007 national census conducted by the Central Statistical Agency of Ethiopia (CSA), this town had a total population of 25,913, of whom 13,035 were men and 12,878 women. Most (97.92%) inhabitants practice Ethiopian Orthodox Christianity, and 2.08% are Muslim. Moreover, 99.45% are members of the Amhara ethnic group. Three governmental high schools currently enroll 4,055 female students, but there are no private schools. There is one primary hospital and two health centers in the district which serve the community. A school-based cross-sectional study design was employed from March 25 to April 17, 2023 in Finote Selam town high school students. The source population was high school students who have been studying in Finote Selam town, and the study population comprised all high school students who were available during the data collection period in 2023.

#### Inclusion criteria

Students who have attended a class during the data collection time were included.

#### Exclusion criteria

High school students who were in serious conditions like illness at data collection time were excluded. Students who transferred from other schools to Finote Selam High School in the second semester of 2023 were also excluded.

### Sample size determination and sampling techniques

The sample size was determined by assuming a single proportional formula. The prevalence of premenstrual dysphoric disorder was taken from a previously published study in Ethiopia at Ayder High School, Mekelle, and the magnitude of premenstrual dysphoric disorder was at 30.9% (22). The sample size (*n*) is calculated using a 95% confidence interval (CI) and a margin of error of 4% as follows:


$$\frac{\text{n}=\left(\text{Zα}/2\right)2\text{p}\left(1-\text{p}\right)}{{\text{d}}^{2}}$$

where n = sample

z = critical value, 1.96

p = assumed prevalence of premenstrual dysphoric disorder from the previous study at Mekelle High School, 30.9%

d = precision (marginal error), 0.04

q = 1 - p

So, the minimum sample size was derived as follows:


$$\frac{\text{n}=\left(1.96\right)2\times 0.309\times 0.691=513}{\left(0.04\right)2}$$

Including 10% of the non-response rate, the final sample size was 513 + 51 = 564.

Associated factors, including severe menstrual pain, high perceived stress, and severe degree of dysmenorrhea, were highly associated with PMDD from previous studies at the University of Gondar and Wollo University (19, 22, 23). Therefore, using Epinfo version 7 software by double population formula, 95%CI and power 80% can be calculated, and high school students in the area of the study were stratified based on their schools in each grade, which are grade 9, grade 10, grade 11, and grade 12. Data from the Education Office indicated that the total number of high school students during data collection was 4,055 (grade 9 = 1,685, grade 10 = 1,070, grade 11 = 778, and grade 12 = 522). Then, the proportional allocation of study subjects for each school and each grade was calculated. Finally, a computer-generated method was used to select study participants from each stratum.

### Data collection tool

Data was gathered using a comprehensive, well-organized questionnaire that was created after evaluating related literature and being adjusted for our situation.

An outcome variable prevalence of premenstrual dysphoric disorder was assessed by using DSM-5. The American Psychiatric Association produced the DSM-5, which is presently used in Ethiopia to diagnose clinical PMDD. Female students were deemed to have PMDD if they reported at least five DSM-5 diagnostic criteria symptoms in most of their menstrual cycles. These symptoms had to be present in the final week before the start of menstruation (1).

The Perceived Stress Scale-10 item (PSS-10), determined to be extremely reliable for assessing the role of stress in the etiology of psychiatric and behavioral illnesses, was used to measure individual stress levels. Scores on the PSS-10 ranging from 0 to 13 indicated low felt stress, scores 14 to 26 indicated moderately perceived stress, and scores 27 to 40 were thought to indicate highly perceived stress. In the evaluation of its dependability, a Cronbach’s alpha of 0.88 was used (24).

Depression in high school was assessed using Patient Health Questioner (PHQ-9). It is a nine-item version, and each item response is rated as “0” (not at all) to “3” (nearly every day); the total score ranges from 0 to 27, with a cutoff ≥5 to indicate having depression symptoms. It has sensitivity of 88% and specificity of 88%. PHQ-9 has been translated and validated in Ethiopia and has been used extensively therein previously to assess depression (25).

The Oslo three-item social support scale, which had a cumulative score range of 3 to 14 and comprised three major categories, was used to evaluate social support. Respondents who scored between 3 and 8, 9 and 12, and 13 and 14 were classified as having weak, moderate, and strong social support, respectively, according to this category. Cronbach’s alpha for the current study was 0.79, indicating acceptable reliability (26).

Structured yes/no questions were used to evaluate clinical factors such as family history of mental illness, history of other mental illnesses, suicidal ideation and attempt, and chronic medical illness.

The World Health Organization’s (WHO) ASSIST (Alcohol, Smoking, and Substance Involvement Screening Test), a highly validated instrument, was utilized in the questionnaire’s behavioral component sections, which include questions about substance use for its evaluation (current use and ever use) (27, 28).

The obstetric and gynecological variables menstrual cycle, menstrual pain, and age of menarche were evaluated using structured questions taken from the literature. The number of pads used per day during the menstrual period was used to calculate the amount of menstruation (22).

Menstrual pain was measured using a three-point verbal rating scale (VRS) with the adjectives mild, moderate, and severe rather than the four-point VRS with a “no pain” category. Only respondents who reported having dysmenorrhea were asked to rate their pain levels (29, 30).

### Data collection

Data were collected using self-administered questionnaires. Three BSc nurses collected data using self-administered questionnaires. Two BSc psychiatry profession supervisors and the principal investigator were participating. For those data collectors and the supervisor, one training day was given before the data collection date. During the training, the objectives of the study were discussed. The data collection methods and tools, as well as how to handle ethical issues, were discussed with the data collectors. The structured questionnaire was also discussed in detail by going through each question with clarification for doubt.

### Data quality assurance

To control the quality of the data, the questionnaire was translated appropriately into the local

Amharic language. At 1 week before the actual data collection, the questionnaire was pretested. Collection time was on 5% (*N* = 28) of Jiga High School students’ studies, which was not be included in the primary survey. Therefore, the dependent variable tool assessment (DSM-5) Cronbach alpha was 0.83. The collected data was adequately handled, reviewed, and checked for completeness and consistency by the supervisor and principal investigator each day.

### Data processing and analysis

The collected data were coded, cleaned, entered, and checked into the computer using EPI data version 4.6.02 and then imported into STATA version 14 to generate descriptive statistics: means, frequencies, percentages, and standard deviations. Logistic regression was used to determine an association between dependent and independent variables and to adjust odds ratios, and the significance level was determined. Using a confidence interval of 95%, univariable and multivariable logistic regressions were used to identify the independent predictors of premenstrual dysphoric disorder. This was done by entering each independent variable separately into the univariable analysis. The variables with a *p*-value of less than 0.2 on the univariable analysis were entered into the multivariable analysis. The statistically significant variables are then considered. An association with a *p*-value less than or equal to 0.05 on logistic regression was considered a predictor of premenstrual dysphoric disorder. Hosmer and Lemeshow test, with a *p*-value of 0.8026 for premenstrual dysphoric disorder, was applied to test the logistic regression model for the goodness of fit. Multicollinearity was performed for the model, and none of the variables scored above 10 (mean VIF = 1.27). For all statistical tests, a *p*-value less than 0.05 was considered significant.

## Results

### Sociodemographic characteristics of the respondents

Data were obtained from 548 female high school students with a response rate of 97.2%. The mean age of the participants was 17.312 ± 1.631, ranging from 15 to 22 years old, and 415 (75.73%) were between 15 to 18 years old. Almost three-fourths, 409 (74.64%), of students were originally from urban areas. The majority of students were single, 469 (85.58%), and more than half of the students lived with their two parents, 316 (57.66%) (**Table 1**).

**Table 1:** Sociodemographic characteristics of the participants among high school students in Finote Selam town (*n* = 548), 2023.

Variables	Category	Frequency	Percent
**Age**	15–18	415	75.73
	>18	133	24.27
**Residence**	Rural	139	25.36
	Urban	409	74.64
**Class of students**	9	226	41.24
	10	143	26.09
	11	107	19.53
	12	72	13.14
**Living arrangement**	Mother and father	316	57.66
	One of the two	62	11.31
	Living alone	84	15.33
	Relatives	52	9.49
	Others	34	6.21
**Mother education level**	Unable to read and write	233	42.52
	Primary	150	27.37
	Secondary	75	13.69
	College and above	67	12.22
	Informal	23	4.20
**Father education level**	Unable to read and write	132	24.08
	Primary	153	27.92
	Secondary	84	15.33
	College and above	131	23.91
	Informal	48	8.76
**Marital status**	Single	462	84.30
	Married	62	11.33
	Divorce	13	2.36
	Widowed	11	2.010
**Absenteeism**	Yes	391	71.35
	No	157	28.65
**Academic performance (score) first semester**	A low score (<70)	266	48.54
	Average score (70–84.99)	179	32.65
	A good score (≥85)	103	18.81
**Pocket money/months**	Yes	109	19.89
	No	439	80.11

Others: home worker, friends, guardian.

### Clinical characteristics of the respondents

Out of the total participants,12 (2.19%) students had a known chronic medical illness, 19 (3.47%) had a history of mental illness, 47 (8.58%) had a family history of mental illness, 31 (5.66%) had lifetime suicide ideation, and 13 (2.32%) students had suicidal ideation within the last 12 months—from those students, seven (1.28%) had a suicide attempt within the last 3 months—and 209 (38.14%) students had depression symptoms (**Table 2**).

**Table 2:** Clinical characteristics of the participants among high school students at Finote Selam town (*n* = 548), 2023.

Variables	Category	Frequency	Percent
**Known chronic medical illness**	Yes	12	2.19
	No	536	97.81
**Diagnosis mental illness**	Yes	19	3.47
	No	529	96.53
**Ever thought of killing**	Yes	31	5.66
	No	517	94.34
**Thought of killing last 12 months**	Yes	13	2.37
	No	535	97.53
**Ever attempt killing self**	Yes	7	1.28
	No	541	98.72
**attempt killing yourself last 12 months**	Yes	5	0.91
	No	543	99.09
**Family history of mental illness**	Yes	47	8.58
	No	501	91.42
**Depressive symptoms**	Yes	209	38.14
	No	339	61.86

### Substance-related factors of the respondents

Regarding substance use, out of out of 548 students, 86 (89.58%) had drunk alcohol at least once in their lifetime, whereas khat and cigarette lifetime users were seven (7.29%) and three (3.13%), respectively. Within the last 3 months, the total number of respondents who used alcohol was 26 (100%) (**Table 3**).

**Table 3:** Substance-related description of the participants among high school students in Finote Selam town (*n* = 548), 2023.

Variables	Category	Frequency	Percent
Ever use of a substance	Yes	96	17.52
	No	452	82.48
Which substance	Alcohol	86	89.58
	Chat	7	7.29
	Cigarette	3	3.13
Within 3 months of ever use of a substance	Yes	26	4.74
	No	522	95.26
Which substance	Alcohol	26	100

### Psychosocial-related factors of the respondents

**Figure 1:**
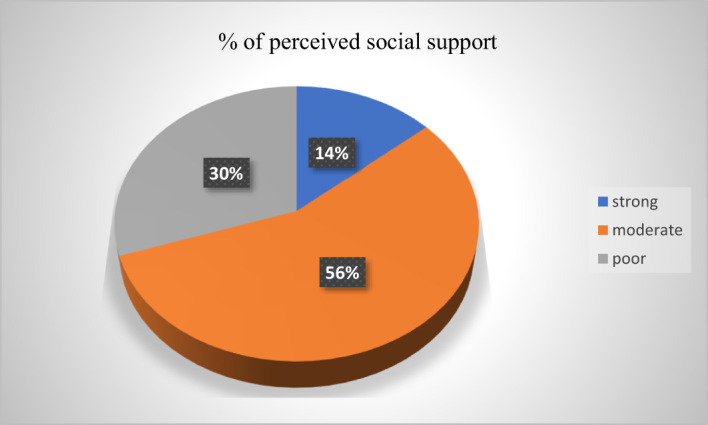
Psycho-social characteristics of participants among high school students in Finote Selam town (*n* = 548), 2023.

Regarding social support factors, among the total participants, 165 (30.11%) had poor social support, 308 (56.20%) had moderate social support, and 75 (13.69%) had strong social support (**Figure 1**).

**Figure 2:**
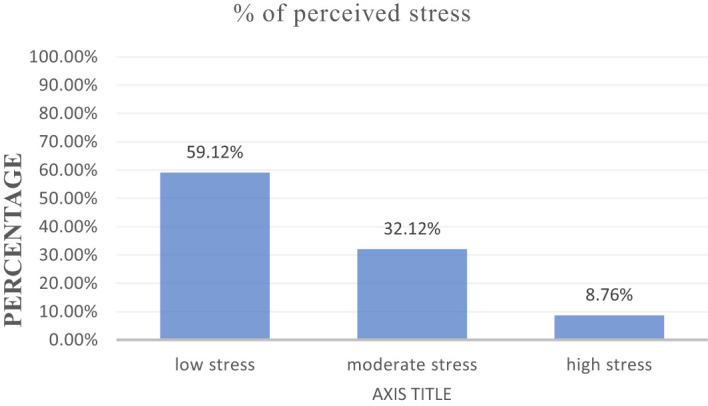
Perceived stress characteristics of low “felt” stress (0 to 13), moderate “felt” stress (14 to 26), and high “felt” stress (27 to 40) among high school in Finote Selam town (*n* = 548), 2023.

Regarding perceived stress factors, from the total participants, 324 (59.12%) had low stress,176 (32.12%) had moderate stress, and 48 (8.7%) had high stress (**Figure 2**).

### Gynecological and obstetric characteristics of the respondents

Of the total participants, 386 (67.15%) had menses that started at the age of 13 to 16 years, and more than half of the respondents, 304 (55.47%), had irregular menstruation periodicity. There were 447 (81.57%) students who reported to have menstrual pain from those, 172 (31.39%) had a long duration of menstruation bleeding, 158 (28.83%) had severe menstrual pain, 214 (39.05%) perceived that the menstrual pain had an impact on their academic performance, and 356 (64.96%) missed their class at least once in their school. A total of 115 (20.99%) respondents had a family history of menstrual-related problems. Of the total respondents, 217 (39.60) used greater than four pads during the menstrual period; those were high amounts of menstrual bleeding. Students who had menstrual pain used different pain management techniques: non-prescribed pain killer for 122 (22.26%), hot drinks for 362 (66.06%), massage for nine (1.64%), and consultation for 55 (10.04%) (**Table 4**).

**Table 4:** Description of the gynecological and obstetric factors of the participants among high school students in Finote Selam town (*n* = 548), 2023.

Variable	Category	Frequency	Percent
**Age when menses start**	Before 13 years	99	18.07
	13 to 16 years	368	67.15
	After 16 years	81	14.78
**Menstruation periodicity**	Regular	244	44.53
	Irregular	304	55.47
**Menstrual pain**	No pain	101	18.43
	Mild pain	175	31.93
	Moderate pain	114	20.81
	Severe pain	158	28.83
**Duration of menstrual bleeding**	≤4 days	376	68.61
	>4 days	172	31.39
**Amount of menstruation**	Minimal (1 pad)	28	5.11
	Moderate (2–4 pads)	303	55.29
	Heavy (>4 pads)	217	39.60
**Family history of menstrual-related problems**	Yes	115	20.99
	No	433	79.01
**Perception of impact on academic performance**	Yes	214	39.05
	No	334	60.95
**Impact of menstrual pain**	Missing classes	356	64.96
	Missing tests	116	21.17
	Decreased score	60	10.95
	Dropped out	16	2.92
**Actions taken for the menstruation pain**	Non-prescribed painkiller	122	22.26
	Hot drinks	354	64.60
	Massage	17	3.10
	Consultation	55	10.04

### Prevalence of premenstrual dysphoric disorder

In this study, the overall prevalence of premenstrual dysphoric disorder among high school students was 33.03% (181) (95%CI: 29.20–37.09). The most commonly reported symptom was physical symptoms such as breast tenderness or swelling, joint or muscle pain, a sensation of “‘bloating,” and weight gain (55.39%), followed by being easily fatigued or a marked lack of energy (48.36%) (**Table 5**).

**Table 5:** Frequency distribution of DSM-5 criteria to assess premenstrual dysphoric symptoms among high school students in Finote Selam town (*n* = 548), 2023.

Variable	Category	Frequency	Percent
Marked affective lability	Yes	186	33.94
	No	362	66.06
Marked irritability or anger or increased interpersonal conflicts	Yes	160	29.20
	No	388	70.80
Marked depressed mood, feelings of hopelessness or self-deprecating thoughts	Yes	167	30.47
	No	381	69.53
Marked anxiety, tension, feelings of being keyed up or on edge	Yes	221	40.33
	No	327	59.67
Decreased interest in usual activities (e.g., work, school, friends and hobbies	Yes	264	48.18
	No	284	51.82
Subjective difficulty in concentration	Yes	260	47.45
	No	288	52.55
Lethargy, easily fatigued, or a marked lack of energy	Yes	265	48.36
	No	283	51.64
A marked change in appetite, overeating, or specific food cravings	Yes	220	40.15
	No	328	59.85
Hypersomnia or insomnia	Yes	119	21.72
	No	429	78.28
Sense of being overwhelmed or out of control	Yes	154	28.10
	No	394	71.90
Physical symptoms such as breast tenderness or swelling, joint or muscle pain, a sensation of “bloating,” weight gain	Yes	303	55.29
	No	245	44.71

DSM-5, Diagnostic and Statistical Manual of Mental Disorders, fifth edition.

### Factors associated with premenstrual dysphoric disorder

In a univariable logistic regression analysis, the factors that fulfilled a *p*-value less than 0.2 were starting age of menses at 13 to 16 years old, menstrual pain, amount of menstruation bleeding, duration of menstruation bleeding days, menstruation periodicity, family history menstruation related problem, perception impact of menstruation pain, perceived stress, having depression symptoms, and treatment seeking behavior. Finally, a multivariable analysis revealed that irregular menstrual cycle, having depressive symptoms, high perceived stress, and duration of menstruation bleeding greater than 4 days were found to be significantly associated with PMDD with 95%CI and at a *p*-value less than or equal to 0.05.

Those who had an irregular menstruation cycle were about (AOR = 4.242, 95%CI = 2.182–8.246) four times more likely to develop PMDD compared with those who had a regular menstruation cycle, and students who had depressive symptoms were about (AOR = 5.272, 95%CI = 2.779–10.002) five times to develop PMDD when compared to students who had no depression. Another associated factor with PMDD was having greater than 4 days of menstruation duration, which is (AOR = 2.138, 95%CI = 1.105, 4.138) two times more odds of having PMDD than those who had less than 4 days of menstruation duration. Students who had high perceived stress were about (AOR = 3.468, 95%CI = 1.217–9.880) 3.5 times to develop PMDD compared to those with low perceived stress (**Table 6**).

**Table 6:** Overall bivariable and multivariable logistic regression analysis of factors associated with premenstrual dysphoric disorder among high school students in Finote Selam town (*n* = 548), 2023.

Variable	Category	PMDD		COR and 95%CI	AOR and 95%CI	P-value
		Yes	No			
**Amount of menstruation bleeding**	Heavy (>4 pad)	151	66	4.829 (2.076, 11.234)	3.095 (0.951, 2.065)	0.060
	Moderate (2–4 pads)	21	282	0.157 (0.063, 1.016)	0.201 (0.059, 1.068)	0.781
	Minimal (1 pad)	9	19	1	1	
**Perceived stress**	High stress	26	22	4.709 (2.509, 8.837)	3.468 (1.217, 9.880)	0.020*
	Moderate stress	90	86	4.169 (2.790, 6.230)	1.197 (0.609, 2.354)	0.601
	Low stress	65	259	1	1	
**Menstruation pain**	Severe pain	114	44	7.876 (4.453, 13.931)	2.259 (0.949, 5.376)	0.149
	Moderate pain	30	84	1.085 (0.587, 2.007)	0.378 (0.150, 1.009)	0.217
	Mild pain	12	163	0.223 (0.106, 1.070)	0.126 (0.045, 1.316)	0.215
	No pain	25	76	1	1	
**Menstruation cycle**	Irregular	148	156	6.066 (3.944, 9.328)	4.242 (2.182, 8.246)	0.000***
	Regular	33	211	1	1	
**Age at menarche**	Before 13 years	16	83	0.550 (0.265, 1.143)	0.403 (0.137, 1.186)	0.082
	13 to 16 years	144	224	1.836 (1.071, 3.149)	2.091 (0.870, 5.026)	0.099
	After 16 years	21	60	1	1	
**Depression**	Depressive	107	102	3.756 (2.584, 5.461)	5.272 (2.779, 10.002)	0.002**
	Not depressive	74	265	1	1	
**Duration of menstruation bleeding**	>4 days	106	66	6.445 (4.329, 9.596)	2.138 (1.105, 4.138)	0.024*
	≤4 days	75	301	1	1	
**Perception impact of pain**	Yes	104	110	3.155 (2.180, 4.567)	0.901 (0.480, 1.691)	0.747
	No	77	257	1	1	
**Family history menstrual related problems**	Yes	57	58	2.448 (1.607, 3.730)	0.996 (0.491, 2.023)	0.993
	No	124	309	1	1	
**Treatment seeking behavior**	Non-prescribed anti-pain	69	53	3.1733 (1.602, 6.283)	0.864 (0.259, 2.876)	0.812
	Hot drink	90	264	0.830 (0.463, 2.623)	0.640 (0.215, 1.901)	0.422
	Massage	6	11	1.329 (0.035, 2.638)	0.163 (0.008, 3.266)	0.236
	Consultation	16	39	1	1	

Hosmer–Lemeshow test = 0.8026, mean VIF = 1.27, Cronbach alpha = 0.725.

*p-value ≤ 0.05; **p-value ≤ 0.01; ***p-value ≤ 0.001.

The bold text indicated that these factors were significantly associated with PMDD.

## Discussion

Premenstrual dysphoric disorder has a negative impact on social interaction and educational achievement by increasing school absenteeism. Because of premenstrual symptoms, students prevent the participants from going to school due to their ignorance of menstruation, and women experience a range of feelings during menarche, including fear, embarrassment, and guilt (31). Those symptoms persist for days to a week: breast tenderness or swelling, joint or muscle pain, a sensation of “bloating,” weight gain, easily fatigued or a marked lack of energy, and difficulty in concentrating. As a result, it is important to ascertain the prevalence of PMDD and identify the risk factors for it. Additionally, this would aid in problem prevention and the development of treatment plans that support female high school students’ academic success.

This study found that the prevalence of PMDD among female high school students was 33.03% (95%CI: 29.20—37.09). This is consistent with other studies done in Ethiopia reported to be 30.9% in Mekelle High School (6) and 34.7% in the University of Gondar (19). Another study in Africa was consistent with this study at Nigeria University (36.1%) (5).

However, the prevalence of premenstrual dysphoric disorder in this study was higher than the previous research findings in Ethiopian students whose prevalence at Debr Berhan University was 13.8% (30) and at Assosa Technical Training College was 26.8% (32). The study’s variation may be due to differences in the study population, with younger individuals more likely to experience premenstrual dysphoric disorder, while older students are less susceptible (33).

The current premenstrual dysphoric disorder prevalence was also higher in studies done in Egypt University (21.1%) (10) and South Africa University (10.2%) (34). The discrepancy might be the difference in the study population as well as sociocultural and socioeconomic characteristics. In Ethiopia, high school students reach puberty and start menstruating often without adequate information and have no psychological readiness to manage it, causing the onset of menstruation (menarche) to be shocking for girls. In high school, the lack of information is accompanied by a lack of access to appropriate sanitary wear and proper facilities for managing menstruation (35).

The finding of the current study was also higher in studies conducted at Kuwait University (5.6%) (36), at Jordan University (7.7%) (37), in Vietnam high schools (1.0%) (38), in Korea (2.4%) (39), in India high schools (4.89%) (40), in Japan high schools (3.1%) (41), among Iran high school students (12.22%) (42), and in Germany (5.8%) (43) as well as with American rates of 6% and 4.7% PMDD, respectively (44, 45). The possible reason for the variation may be differences in socioeconomic determinants. This means that, in developed countries, there are good sanitary products, adequate water supply, and privacy for changing sanitary pads, which continue to leave high school students unlimited options for safe and sufficient menstrual hygiene in high-income settings. Because of this reason, female students in menstruation will feel less shame, self-isolation, irritability, emotional liability, headaches, anxiety, depression, and traumatic experiences (46). Furthermore, the availability of health facilities between those countries and Ethiopia could be poor due to poor healthcare infrastructure and a shortage of trained health staff, which would lead to the delivery of inadequate healthcare services. In turn, premenstrual dysphoric disorder might not be identified and treated early.

In terms of sociocultural aspects, in Ethiopia, there is also a common belief (more commonly held by men) that menstruation does not start until a girl has a sexual intercourse for the first time (31). The existence of this false belief presents a potential hazard for girls approaching menarche if they live in families where this belief is held. The beginning of menses in girls is manifested when irritability, emotional liability, headaches, anxiety, depression, and traumatic experiences appear.

The other reason is that menstrual hygiene management is influenced by a variety of factors, including knowledge of reproductive biology, background beliefs about menstruation that are prevalent in societies, and the limitations that these beliefs place on female-related activities. Due to their confusion, embarrassment, and lack of preparation for the abrupt and unexpected onset of menstruation, adolescent girls may find it difficult to grow academically and socially due to menstrual hygiene management issues (47, 48). In terms of the type of study populations, between high school and university students, the latter have increased their knowledge about menstruation and experience of menses and thus have decreased behavioral changes due to menstruation.

On the other hand, the current study’s finding is lower than the previous study done at Ethiopian Wollo University (66.9%) (21). The discrepancy may be the fact that the study at Wolo University surveyed first year to fourth year students, primarily first year students. As students transition into higher education, they face challenges like integrating into university culture, socioeconomic issues, interpersonal problems, demanding course loads, and insufficient institutional support, making them vulnerable to stress and depression (49), which might increase the prevalence of PMDD.

The current study’s finding was also lower in studies conducted at Morocco University (55%) (50). The discrepancy might be due to the tools of assessment used in Morocco. The Moroccan high school students used the Daily Record of Severity of Problems (DRSP), but DRSP is a screening tool and not a diagnostic tool, and this might overestimate the prevalence of premenstrual dysphoric disorder among students (51). Moreover, the other discrepancy might be the sociocultural aspect since students in Ethiopia do not express their actual feelings related to premenstrual symptoms such as depressed mood, irritability, emotional, and somatic symptoms because of the culture of silence and associated stigma around menstruation; thus, this topic is not openly discussed at the family level. The abovementioned reason might be the reason for the lower prevalence of PMDD in this study (33).

Regarding factors affecting premenstrual dysphoric disorder, the duration of menstruation bleeding was significantly associated with a higher rate of premenstrual dysphoric disorder. The odds of premenstrual dysphoric disorder were two times among those students having longer durations of menstruation bleeding than students with shorter durations (<4 days). These findings were supported by other studies done among Mekelle high school students, Ethiopia (6), and among university students from Egypt (10) and Nigeria (5). The possible reason might be that having a long duration of menstruation bleeding days could cause fluctuations ranging from hormone-related issues like estrogen and progesterone that can increase the vulnerability to premenstrual dysphoric disorder because a drop in estrogen and progesterone may lead to a reduced level of serotonin, which can result in increased levels of sadness, anxiety, and irritability when women are on menses. Other reasons might be that, with a long duration of menstruation bleeding, students become absent from school and have decreased social interaction, which can increase the academic burden on students and which, in turn, can lead them to have low self-confidence and poor academic performance (31, 35, 52).

The present study also showed that premenstrual dysphoric disorder is significantly associated with high perceived stress among high school students. The odds of premenstrual dysphoric disorder were more than three times higher among students with high perceived stress than those without such stress. Similar findings were reported in the University of Gondar, Ethiopia, and Jordan (19, 53). The possible reason might be the development of anxiety and depressive symptoms like tension, a sense of difficulty in controlling one’s self, a depressed mood, and irritability resulting from people becoming more stressed, thus impairing their ability to concentrate in school.

The aggravation of cardinal mood symptoms may be caused by premenstrual symptoms like anger and irritability which are linked to stress-related premenstrual severity. Others claim that students with low-income levels had a higher perceived stress, which can indirectly affect the prevalence of PMDD compared to those with higher incomes. Furthermore, this may be due to believing that having a low income is a very stressful situation in life, making premenstrual symptoms more severe and prevalent (54, 55).

Students with irregular menstrual cycles had about four times higher odds of premenstrual dysphoric disorder than students with regular menstrual cycles. This finding is consistent with other findings at Assosa Technical Training College, Ethiopia (32). Students who experience irregular menstrual cycles may develop a premenstrual dysphoric disorder for various reasons, i.e., students may experience high rates of absenteeism from class due to irregularities of menstruation each month, premenstrual symptoms, a lack of attention to their problems, and a lack of confidence during bleeding, all impacting their capacity to learn (56).

Students with depressive symptoms had about five times higher odds of premenstrual dysphoric disorder as compared with those who had no depressive symptoms. This finding is consistent with findings from Nigeria University (5) and among Korean women (57). The possible reason could be that women have more depressive symptoms, such as different somatic and affective symptoms that interrupt their function, which can aggravate those somatic and affective symptoms like joint pain, irritability, lack of energy, depressed mood, and difficulty in concentrating during menstruation (58).

### Strength and limitation of the study

We used an adequate sample for the study using an appropriate probability sampling technique and data collection procedure. However, there is recall bias—for example, age when menses started—, and the cross sectional study design by itself cannot assess the cause and effect relationship.

## Conclusion

In this study, the magnitude of premenstrual dysphoric disorder was one-third among high school students, and it was higher compared to other studies. Long-duration menstruation periods, irregular menstruation cycles, high perceived stress, and depressive symptoms were factors significantly associated with PMDD among students. Therefore, it needs early screening and intervention in primary healthcare settings, especially for those who have high perceived stress, depressive symptoms, a long duration of menstruation bleeding, and an irregular menstruation cycle, so as for them to have good academic achievement and psychological wellbeing. Before menstrual symptoms can have an impact on students’ academic performance, early diagnosis and intervention are required. Stress reduction programs could be a useful non-pharmaceutical treatment for relieving psychological and physical symptoms.

## Data availability statement

The raw data supporting the conclusions of this article will be made available by the authors, without undue reservation.

## Ethics statement

The studies involving humans were approved by Ethical review committee of the University of Gondar. The studies were conducted in accordance with the local legislation and institutional requirements. Written informed consent for participation in this study was provided by the participants’ legal guardians/next of kin.

## Author contributions

GK: Writing – review & editing, Writing – original draft, Visualization, Supervision, Project administration, Methodology, Formal analysis, Conceptualization. DD: Software, Supervision, Writing – review & editing. SK: Writing – review & editing, Supervision, Software. SY: Writing – review & editing, Visualization, Supervision, Software. MK: Writing – review & editing, Visualization, Supervision, Investigation, Formal analysis. MM: Writing – review & editing, Methodology. GT: Writing – review & editing, Visualization, Supervision, Formal analysis. SF: Writing – review & editing, Visualization, Supervision, Software, Formal analysis. GN: Writing – review & editing, Visualization, Supervision, Software, Formal analysis. YW: Writing – review & editing, Methodology, Supervision, Visualization. TA: Writing – review & editing, Visualization, Methodology, Investigation, Formal analysis.
